# Case report: Control of intestinal nematodes in captive *Chlorocebus sabaeus*

**DOI:** 10.4102/ojvr.v88i1.1903

**Published:** 2021-05-28

**Authors:** Katalina Cruz, Tatiana M. Corey, Michel Vandenplas, María Trelis, Antonio Osuna, Patrick J. Kelly

**Affiliations:** 1 Faculty of Veterinary Medicine, Ross University School of Veterinary Medicine, Basseterre, St Kitts and Nevis, West Indies; 2 Department of Parasitology, Faculty of Sciences, Institute of Biotechnology, Biochemistry and Molecular Parasitology, University of Granada, Granada, Spain; 3 St Kitts Biomedical Research Foundation and Virscio, St Kitts and Nevis, Lower Bourreyaeu, West Indies; 4 Department of Parasitology, Faculty of Pharmacy, University of Valencia, Valencia, Spain; 5 Research Unit on Endocrinology, Nutrition and Clinical Dietetics, Health Research Institute La Fe, Valencia, Spain

**Keywords:** *Capillaria*, *Trichuris*, hookworm, *Trichostrongylus*, *Strongyloides*, albendazole, ivermectin, captive, African green monkeys

## Abstract

There are limited data on the efficacy of antiparasitic treatments and husbandry methods to control nematode infections in captive populations of African green monkeys (AGMs), *Chlorocebus sabaeus*. In faecal egg count (FEC) tests, 10 of the 11 (91%) adult male AGMs captured from the large feral population on the island of St Kitts had evidence of nematode infections, mostly *Capillaria* (8/11, 73%), *Trichuris trichiura* (7/11, 64%) and strongylid species (7/11, 64%) specifically (hookworm and *Trichostrongylus*, 50/50), but also *Strongyloides fuelleborni* (1/11, 9%). When kept in individual cages with cleaning and feeding regimens to prevent reinfections and treated concurrently with ivermectin (300 µg/kg, given subcutaneously) and albendazole (10 mg/kg, given orally) daily for 3 days, 60% (6/10) of the AGMs were negative at a follow-up FEC at 3 months and by FEC and necropsy at the end of the study 5–8 months later. One monkey appeared to have been reinfected with *T. trichiura* after being negative by FEC at 3 months post-treatment. Four AGMs were positive for *T. trichiura* at the 3 month FEC follow-up but were negative at the end of the study after one further treatment regimen. Although initially being cleared of *Capillaria* following treatment, three AGMs were found to be infected at the end of the study. The ivermectin and albendazole treatment regimen coupled with good husbandry practices to prevent reinfections effectively controlled nematode infections in captive AGMs.

## Introduction

*Chlorocebus sabaeus*, African green monkeys (AGMs) of the Cercopithecidae family, widely found in Africa, were introduced onto the Caribbean island of St Kitts in the 17th century (McGuire 1974). There are limited published data on the parasitic burden of the vast (perhaps 50 000; Dore, Gallagher & Mill 2021 – unpublished) feral population of AGMs, which commonly come into contact with people and may play a role in the epidemiology of human zoonotic parasitic infections common on the island (Berger 2020). Incomplete Caribbean studies have reported island AGMs to be infected with *Trichuris trichiura* (Yao et al. 2018), *Strongyloides* sp. (Gallagher et al. 2019; Ritchie et al. 1967), *Primasubulura* sp. (Cameron 1930) and *Schistosoma mansoni* (Cameron 1928).

There are limited data on effective anthelmintic treatments for AGMs and husbandry recommendations to control parasitic infections in captive research animals. Nonhuman primate research facilities require effective parasite treatment and prevention protocols to minimise infections that may confound research outcomes and cause unnecessary stress, which lowers the quality of life of study animals. Ritchie et al. (1967) reported that thiabendazole [two 50 mg/kg doses, PO 14 days apart] significantly reduced or eliminated *Strongyloides* eggs in the faeces. Kagira et al. (2011) reported that a 3-day regimen of albendazole (7 mg/kg, PO) and ivermectin (300 µm/kg, SQ) reduced the faecal egg counts (FEC) of *T. trichiura* and strongyles consistently by 100% after 7, 14 and 28 days. Chapman et al. (2016) reported that two doses of ivermectin (300 µg/kg, PO) 5 days apart resulted in a 100% cure rate for nematodes in wild AGMs, with no eggs found in the faeces 1 month post-treatment.

Unfortunately, the evaluation of nematode infections is challenging because of inadequate data on the drug efficacy in AGMs and the lack of studies investigating prolonged follow-up, which is needed to detect confounding factors (autoinfection, reinfection, larval transplacental or colostrum transmission, dormant stages and autoinfection resulting from parthenogenesis) (Reichard et al. 2017). Furthermore, FECs can be unreliable, and studies that enable necropsy evaluations are relevant in the absence of a ‘gold standard’ (Geary et al. 2010).

In this report, we provide a more complete description of the nematode parasites present in AGMs on St Kitts and evaluate a treatment protocol and husbandry practices to control infections in captive AGMs.

## Case report

### Animals and facilities

The St Kitts Biomedical Research Foundation (SKBRF) and translational research affiliate Virscio, Inc. are American Association of Accredited Laboratory Animal Care (AAALAC)-accredited biomedical research facilities for integrated preclinical research and development with a multidisciplinary non-human primate focus. The facility houses around 1000 *C. sabaeus* study animals according to regulations in the *Guide for the Care and Use of Laboratory Animals* (National Research Council of the National Academies 2011).

The convenience sample of apparently healthy AGMs used in our study were enrolled in other terminal studies that had been approved by the Institutional Animal Care and Use Committee (approval number AC18175) and conducted in 2019. These studies did not involve procedures that would have influenced the parasitological status of the animals. Eleven wild-caught adult male AGMs were housed in individual stainless steel cages that meet non-human primate requirements (National Research Council of the National Academies 2011) for the duration of their study period. The cages were washed down with water twice daily, and every 2 weeks they were sanitised using a combination of steam (82 °C) and a quaternary-based disinfectant (Consume Eco-Lyzer, Consume Nature’s Way, Spartan Chemical Company Inc., Ohio, Maumee, United States [US]), or an accelerated hydrogen peroxide foam (Peroxigard, Virox Technologies Inc., Oakville, Ontario, Canada). Adenosine triphosphate bioluminescence testing of randomly selected dry surfaces was used to determine the sanitation effectiveness.

The main food provided was monkey chow (Envigo Teklad 8773 primate biscuits, Indiana, Indianapolis, US) once a day supplemented with seasonal produce sourced locally. The produce was provided for both nutrition and as an enrichment activity for the animals’ well-being, and it was washed first if evidently soiled. Water, provided *ad libitum* with Lixit water valves, was filtered (Neo-Pure PS-27097-05 9 3/4 inch, 5 microns, NeoLogic Solutions, Greenville, South Carolina, US) and treated with ultraviolet light (Sanitron ultraviolet water purifiers, Atlantic Ultraviolet Corporation, Hauppauge, New York, US). The water lines were sanitised monthly with a sodium hypochlorite flush delivered by an Edstrom automated watering system (Avidity Science, Waterford, Wisconsin, US).

### Treatments

The deworming regimen was based on a report from Kenya (Kagira et al. 2011) and consisted of ivermectin (300 µg/kg, SQ; Noromectin, Norbrook, Newry, Co. Down, Northern Ireland) and albendazole (IVAL) (10 mg/kg, PO by nasogastric or orogastric gavage; Valbazen, Zoetis, Kalamazoo, Michigan, US) daily for 3 days, following chemical restraint (ketamine HCl, 8 mg/kg, Bioniche Pharma USA LLC, US, and xylazine, 1.6 mg/kg, AnaSed, Akorn Inc., Lake Forest, Illinois, US). The animals were returned to their cages and monitored by cage-side observation twice a day for the duration of the study period. No side effects were observed post-treatment. The treatment was repeated if an animal had a positive FEC result during the routine 3-month screening.

### Monitoring of treatments

Day 0 and 3 month follow-up FECs were carried out on faeces collected directly from the cages. A final FEC and counts of adult *T. trichiura* in the large intestine were performed at routine necropsies 6 or 8 months after the initial IVAL treatment. For the recuperation of adult nematodes, the large intestine was carefully examined whilst removing the ingesta with a circular rubbing motion, washed with 0.9% saline, filtered through a 100-µm sieve and examined for nematodes under a stereomicroscope (7× – 10× magnification).

### Faecal analysis and morphologic identification

**FIGURE 1:**
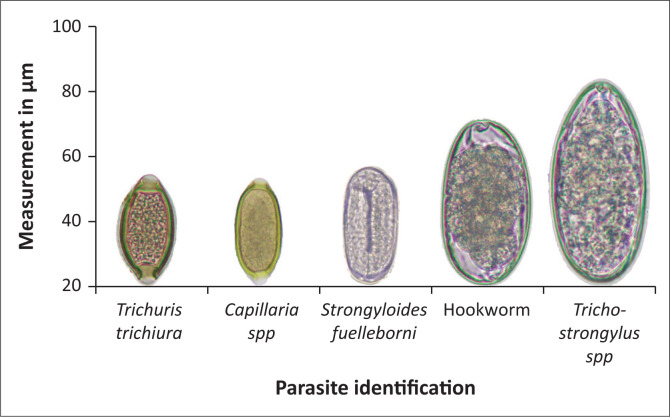
Morphologic identification of nematode egg species found (measurements in micrometres).

For FECs, aliquots (2 g) of uncontaminated faeces were collected from the cage’s under-tray and analysed within 48 h using the double centrifugation technique (5 min, 500 g) with Sheather’s sugar solution (spg 1.28). Eggs were identified to the species level whenever possible using described morphological characteristics (Modrý et al. 2015) (Table 1 and Figure 1).

**TABLE 1:** Morphologic identification guidelines followed for egg identification.

Species	Size (µm)	Colour	Shape	Shell	Developmental stage
*Trichuris trichiura* eggs	50–60 × 20–30	Brown-bile green	Lemon-shaped with bipolar plugs	Smooth	Unembryonated.
*Capillaria* eggs	50–60 × 20–30	Light yellow/green	Ovoid barrel-shaped with bipolar prominences	Characteristic striated shell	Unembryonated.
*Strongyloides fuelleborni* eggs	50–60 × 30–40	Colourless appearance	Ellipsoidal	Thin shell	U-shaped larvae (L1) inside the egg shell.
Strongylid eggs	60–85 × 35–50	Colourless appearance	Ellipsoidal	Smooth and thin shelled	Hookworms: morula and smaller in size. *Trichostrongylus* spp.: early cleavage-stage embryo, one or both eggshell ends more pointed, slightly longer.

*Source*: Modrý, D., Petrželková, K.J., Kalousová, B. & Hasegawa, H., 2015, *Parasites of African Great Apes, Atlas of coproscopic diagnostics*, HPI-Lab, Brno.

### Ethical considerations

The African green monkeys used in our study were enrolled in other terminal studies that had been approved by the Institutional Animal Care and Use Committee of the St Kitts Biomedical Research Foundation. Approval to conduct the study was also obtained from St. Kitts Biomedical Research Foundation, Virscio and Axion Research Foundation, Biomedical Research of the Central Nervous System (IACUC AC18175).

## Results and discussion

Ten of the eleven (91%) wild-caught adult *C. sabaeus* study subjects, all male, employed in the study were infected with a variety of nematodes: *Capillaria* (8/11, 73%), *T. trichiura* (7/11, 64%), strongylids (7/11, 64%; hookworms and *Trichostrongylus* [50/50]) and *Strongyloides fuelleborni* (*S. fuelleborni*) (1/11, 9%) (Tables 2 and 3). Whilst the latter species have been described in AGMs in Africa previously (Chapman et al. 2016; Gallagher et al. 2019; Yao et al. 2018), our finding of *Capillaria* on the island is of note as eggs of this genus have not previously been reported in the faeces of AGMs.

**TABLE 2:** Faecal egg count results and adult *Trichuris trichiura* found at necropsy in animals treated with ivermectin and albendazole.

Animal number	Pretreatment FEC				FEC 3 months after one IVAL treatment				FEC 6–8 months after one IVAL treatment				*T. trichiura*observed in the large intestine at necropsy 6–8 months after one IVAL
	*Capillaria*	*T. trichiura*	Strongylids	*S. fuelleborni*	*Capillaria*	*T. trichiura*	Strongylids	*S. fuelleborni*	*Capillaria*	*T. trichiura*	Strongylids	*S. fuelleborni*	
**1**	−	+	+	−	−	−	−	−	−	−	−	−	−
**4**	−	+	+	−	−	−	−	−	−	−	−	−	−
**6**	+	+	+	−	−	−	−	−	−	−	−	−	−
**7**	+	−	+	−	−	−	−	−	−	+	−	−	+
**8**	−	−	−	−	−	−	−	−	−	−	−	−	−
**10**	+	+	+	−	−	−	−	−	−	−	−	−	−
**11**	+	−	−	−	−	−	−	−	−	−	−	−	−

Note: Animals that tested negative by faecal egg count 3 months after treatment with ivermectin and albendazole were not re-treated whilst those that were positive were redosed with ivermectin and albendazole.

FEC, faecal egg count; IVAL, ivermectin and albendazole; −, negative; + positive; *T. trichiura, Trichuris trichiura; S. fuelleborni, Strongyloides fuelleborni*.

**TABLE 3:** Faecal egg count results and adult *Trichuris trichiura* found at necropsy in animals treated with ivermectin and albendazole.

Animal number	Pretreatment FEC				FEC 3 months after first IVAL treatment				FEC 3 months after second IVAL treatment				*T. trichiura*observed in the large intestine at necropsy 3 months after second IVAL
	*Capillaria*	*T. trichiura*	Strongylids	*S. fuelleborni*	*Capillaria*	*T. trichiura*	Strongylids	*S. fuelleborni*	*Capillaria*	*T. trichiura*	Strongylids	*S. fuelleborni*	
**2**	+	+	+	+	−	+	−	−	+	−	−	−	−
**3**	+	−	−	−	−	+	−	−	−	−	−	−	−
**5**	+	+	−	−	−	+	−	−	+	−	−	−	−
**9**	+	+	+	−	−	+	−	−	+	−	−	−	−

**Total**	**8/11 (73 % )**	**7/11 (64 % )**	**7/11 (64 % )**	**1/11 (9 % )**	**-**	**-**	**-**	**-**	**-**	**-**	**-**	**-**	**-**

Note: Animals that tested negative by faecal egg count 3 months after treatment with ivermectin and albendazole were not re-treated, whilst those that were positive were redosed with ivermectin and albendazole.

FEC, faecal egg count; IVAL, ivermectin and albendazole; −, negative; +, positive; *T. trichiura, Trichuris trichiura; S. fuelleborni, Strongyloides fuelleborni*.

Infections, then, might have only been acquired in the 300 years since AGMs were introduced onto St Kitts. Unfortunately, we could not determine the *Capillaria* species involved as egg morphology alone cannot be used for speciation, and local experience is that no adults have been reported in the necropsy findings of AGMs on the island of St. Kitts.

Overall, the IVAL treatment regimen together with the husbandry measures in place in our study were relatively successful at controlling nematode infections in captive AGMs. Following a single IVAL treatment, 6/10 (60%) of the infected AGMs were negative for nematodes 6–8 months later as confirmed by FEC and at necropsy (Table 2). A second IVAL treatment given to the four AGMs with persistent *T. trichiura* infections 3 months after the first treatment, resulted in the elimination of the *T. trichiura* 3–5 months later as evidenced by negative FECs and necropsy findings (Table 3). Although apparently cleared of *Capillaria* following the first IVAL treatment, three of these four AGMs had *Capillaria* eggs in their faeces at necropsy 3–5 months after a second treatment.

The apparent treatment failures with *T. trichiura* and *Capillaria* described above warrant further comment. We suspect that the four AGMs that had *T. trichiura* eggs 3 months after the first treatment with IVAL most likely had infections in the prepatent period, which might exceed 12 weeks in humans (Bundy & Cooper 1989). It is also possible that the animals became infected whilst in captivity, which is the most likely explanation for the single AGM (Animal 7; Table 2) that was negative for *T. trichiura* 3 months after the first IVAL treatment but was positive for adults and eggs at necropsy after 8 months. *Trichuris* eggs can remain inactive on surfaces for years (Bundy & Cooper 1989; Reichard et al. 2017), and although the AGM cages in our study were cleaned and disinfected regularly, *Trichuris* eggs are difficult to inactivate (Bundy & Cooper 1989). It is of note that six of these seven animals that were negative for FEC 3 months after the one IVAL treatment were still negative 6–8 months post-treatment. This indicates that the hygiene measures in use in our facility were mostly effective at preventing reinfection.

How four AGMs became positive for *Capillaria* following the apparent resolution of infection after the first IVAL treatment is unclear; without being able to determine the infecting specie/s, it is not possible to determine whether these AGMs had been in the prepatent period during treatment, were autoinfected, reinfected or had false-negative test results previously.

The main problem with the IVAL treatment regimen is the requirement for three consecutive sedations for albendazole administration; such repeated dosing, however, is needed for effective ovicidal and larvicidal activity (Martin, Robertson & Bjorn 1997). Further, this also enabled three administrations of ivermectin, which helps overcome the problem that single doses perhaps only temporarily inhibit the motion and feeding of nematodes instead of causing their death or expulsion (Martin et al. 1997).

## Conclusion

African green monkeys can harbour several nematode species, and effective anthelmintic protocols are needed to ensure the optimal health and well-being of animals in captivity and to prevent zoonotic infections in handlers and local people. The IVAL treatment protocol and husbandry practices we describe would appear to be effective in controlling nematode infections in captive AGMs.
